# Deafness from Scarlet Fever1Read at the thirty-fourth annual meeting of the Medical Association of Central New York, held at Buffalo, N. Y., August 27 and 28, 1901.

**Published:** 1901-11

**Authors:** Sargent F. Snow

**Affiliations:** Syracuse, N. Y.


### Deafness from Scarlet Fever.[1]

SARGENT F. SNOW, M. D., Syracuse, N. Y.

THAT the disease, scarlet fever, is responsible for quite a percentage of our incurable cases of deafness is too well known to be questioned. It often also goes further and fastens upon the patient that constant menace to life—a chronic suppurative discharge from the middle-ear with the dangers of

1. Read at the thirty-fourth annual meeting of the Medical Association of Central New York, held at Buffalo, N. Y., August 27 and 28, 1901.

mastoid and intercranial invasion, hence it behooves us by discussion and otherwise to ferret out each possible advance in the prevention and cure of its ravages.

The acute inflammation of the pharynx that accompanies scarlatina differs from the inflammation that occurs in other germ diseases, principally in its degree of activity. It is perhaps too much to expect that we can ever entirely prevent aural involvements because of this activity of the morbid processes in the early part of the disease, but there can be an improvement in the amount of attention given to the throat and ear symptoms.

Extreme impairment of hearing that lasts throughout life often is the result of neglect in the simplest precautionary measures. Perfect drainage and cleanliness are as much indicated in the ear inflammations that occur in scarlet fever and measles as in those that occur from influenza and common colds.

Pain in the ear is a warning of impending disaster. A most destructive necrosis may follow, involving not only the function of hearing but life itself. The organism producing this necrosis in scarlatina possesses much virulence, and if pent up will do lasting damage. Acute meningeal inflammations are often traceable to the middle-ear. No laxity of effort can be excused; local treatment indicated at this time promotes the general recovery. In each case the nasal passages should be sprayed by alkaline antiseptic solution, followed by a soothing oily lotion, and if active ear symptoms do supervene, hot irrigations 110 to 125° Fahrenheit, or tympanic incision and the Sprague ice bag, will not only relieve suffering but materially reduce inflammatory action.

Just in this connection, under the plan of treatment briefly outlined above, nice points of discrimination often arise concerning the advisability of opening the drum head, and the length of time that the ice bag should be kept on; the first can with safety always be answered in the affirmative; a good opening is needed, free drainage hastens, and helps a long way toward a complete result. The second point by the general statement that constant cold applications should be continued as long as they appear to do good.

It has been wisely said that "an ounce of prevention is worth a pound of cure," but in the graver cases of scarlet fever with high range of temperature, much vomiting, details of quarantine and the like, the attending physician is forced to use his later prerogatives. The collection within the middle-ear cavity has perhaps ruptured the drum nature attempted to give relief; if she made the opening free enough, antiseptic cleansing may be all that is necessary—otherwise the best care of a skilled otologist is none too good.

In the consideration of the chronically deaf from this disease, a different proposition is presented. The case is practically beyond the reach of the general practitioner and many times the destructive action has been so great that the specialist can do no good, further than correcting discharges, a procedure very essential and sometimes hard to accomplish. Then again it may be found that the suppurative action has destroyed only a portion of the drum, leaving the ossicular chain in a fairly good condition, in which event no case should be discouraged until attempts at repairing the natural drum or fitting a false one are carefully made. A brief outline of two of my cases will perhaps be interesting in this connection and show what may be accomplished under favorable circumstances.

Case I.—Miss D., referred by Dr. A. S. Edwards, aged 23, general health fair, came to me September 25, 1893, with history of thick watery discharge, with odor from each ear since having scarlet fever when four years old; R.H.D., was 12-100, L.H.D., 3-100 for the watch. Had for a time noticed something growing in the ear and that the discharge was more or less bloody. An examination showed both external canals plugged with pale red masses, which were immediately snared out, and later the stumps treated with chromic acid fused on a probe and carefully applied at different sittings. Along with this treatment, attention was paid to an attending catarrhal state of the nasal and post-nasal membranes, removing overgrown portions of the middle turbinates and otherwise improving the local head conditions with the result that on March 22, 1895, thirty-two months after beginning treatment, the perforation in each drum was closed and her hearing by same tests was 100-100, and was still normal the last time I saw her July 5, 1901.

Case II.—Mrs. A., aged 29, referred by Dr. R. C. McLennan, with a history similar to the one just mentioned except that there had been no bloody character to the discharge, though it had been continuous and foul since having the fever when six years old. Examination showed the very common state, pustular discharge, the whole drum and ossicular chain destroyed in one ear and a large perforation in the drum of the other. The hearing in both ears was so much diminished that watch sounds could not be appreciated, but numbers spoken in ordinary voices she could hear by the left ear, six feet.

Treatment along the line above described succeeded in correcting the discharge from the left ear, (the one that had not suffered such extensive ravages) and later, after reducing a catarrhal sensitiveness that existed in the post-nasal space and eustachian tube on that side, a false drum made from a disk of rubber film was fitted: The hearing immediately improved to 24 feet for ordinary voice sounds, and 86 inches for the forced whisper, a gain of 400 per cent., which holds good at the present time. The discharge from the right ear, though very thin and light, has never absolutely ceased owing to a small area which has resisted such curettement as can be given without a general anesthetic. Neither has the hearing on that side changed, but the improvement in the left ear has been so marked that we are perfectly satisfied.

Personal experience has taught me that minute applications of chromic acid to the stumps of polyps or to granulated areas, as described above, are very effective in correcting chronic discharges where nitrate of silver has failed, but they must be made by a very steady hand and under good illumination, touching only the proper tissues. Repeated stimulation of the edges, if the hole be not too large, is well recognised as being quite reliable in healing over old perforations in the tympanum.

A practical point in any and all cases that I would again emphasize is the correction of catarrhal tendencies in the head membranes; no artificial appliance will be tolerated nor improvement be permanent if the condition be repeatedly aggravated by inflammation from colds, or nasal malformations.

The false drum mentioned differs from the one commonly used, as a thread takes the place of the steel wire shank, thereby lessening the friction sounds and chances of irritation. As described by Greuber (see page 291 of his work on Diseases of the Ear), the drum is inserted by the introducer, which is simply a piece of small steel wire one inch long, with small eyelet at one end bent at a right angle, through which the thread is passed, holding the rubber film firmly against the eyelet until placed in the ear, when the wire introducer is withdrawn, the excess thread pushed into the canal and left from one to three days.

These two cases selected from my records are simply representative of a large class that come to the otologist and just such ones that are going through life with not even an examination by an expert to see whether the destructive action has been so great that there is no help in either of the above methods.

The popular opinion that a cloak of mystery surrounds the ear condition in or following scarlet fever is wrong, and should in no instance shield a lack of professional effort. Aggressive scientific work pays well.

707-709 University Block.

